# Complexity of options related to restarting oral poliovirus vaccine (OPV) in national immunization programs after OPV cessation

**DOI:** 10.12688/gatesopenres.14511.1

**Published:** 2023-04-17

**Authors:** Dominika A Kalkowska, Steven GF Wassilak, Eric Wiesen, Concepcion F Estivariz, Cara C Burns, Kamran Badizadegan, Kimberly M Thompson

**Affiliations:** 1 Kid Risk, Inc., Orlando, FL, USA; 2 Global Immunization Division, Center for Global Health, Centers for Disease Control and Prevention, Atlanta, GA, USA; 3 National Center for Immunization and Respiratory Diseases, Centers for Disease Control and Prevention, USA, Atlanta, GA, USA

**Keywords:** polio, eradication, vaccine, dynamic modeling

## Abstract

**Background:** The polio eradication endgame continues to increase in complexity.  With polio cases caused by wild poliovirus type 1 and circulating vaccine-derived polioviruses of all three types (1, 2 and 3) reported in 2022, the number, formulation, and use of poliovirus vaccines poses challenges for national immunization programs and vaccine suppliers.  Prior poliovirus transmission modeling of globally-coordinated type-specific cessation of oral poliovirus vaccine (OPV) assumed creation of Sabin monovalent OPV (mOPV) stockpiles for emergencies and explored the potential need to restart OPV if the world reached a specified cumulative threshold number of cases after OPV cessation.

**Methods:**  We document the actual experience of type 2 OPV (OPV2) cessation and reconsider prior modeling assumptions related to OPV restart.  We develop updated decision trees of national immunization options for poliovirus vaccines considering different possibilities for OPV restart.

**Results:**  While OPV restart represented a hypothetical situation for risk management and contingency planning to support the 2013-2018 Global Polio Eradication Initiative (GPEI) Strategic Plan, the actual epidemiological experience since OPV2 cessation raises questions about what, if any, trigger(s) could lead to restarting the use of OPV2 in routine immunization and/or plans for potential future restart of type 1 and 3 OPV after their respective cessation.  The emergency use listing of a genetically stabilized novel type 2 OPV (nOPV2) and continued evaluation of nOPV for types 1 and/or 3 add further complexity by increasing the combinations of possible OPV formulations for OPV restart.

**Conclusions:** Expanding on a 2019 discussion of the logistical challenges and implications of restarting OPV, we find a complex structure of the many options and many issues related to OPV restart decisions and policies as of early 2023.  We anticipate many challenges for forecasting prospective vaccine supply needs during the polio endgame due to increasing potential combinations of poliovirus vaccine choices.

## Introduction

Global polio endgame modeling ^1^ recognized the varied and complex decisions that national immunization program leaders face ^2– 6^ when considering the different properties, risks, and benefits of the two major categories of poliovirus vaccines: oral poliovirus vaccine (OPV) and inactivated poliovirus vaccine (IPV) ^1, 7– 9^. Successfully ending and preventing the transmission of wild polioviruses (WPVs) and/or circulating vaccine-derived polioviruses (cVDPVs) requires achieving high population immunity to transmission ^10^ using polio vaccines delivered in routine immunization (RI), and supplementary immunization activities (SIAs) in areas with low RI coverage, such that at one point in time all circulating polioviruses die out. However, despite its safety and effectiveness, the use of Sabin OPV poses low, but non-zero, risks of vaccine-associated paralytic polio (VAPP) and polio cases caused by vaccine-derived polioviruses (VDPVs) ^7, 11^. Consequently, as part of the polio endgame, the Global Polio Eradication Initiative (GPEI) identified the need to stop all OPV use to end all cases of poliomyelitis ^12, 13^, which it decided to implement using a phased approach by first stopping all type 2 OPV use (OPV2) ^14^. Notably, the last reported case of polio caused by type 2 WPV (WPV2) transmission occurred in 1999 and certification of eradication of indigenous WPV2 transmission occurred in 2015 ^15^ in preparation for globally-coordinated OPV2 cessation in 2016.

Many modeling studies of poliovirus transmission dynamics showed that high quality (i.e., rapid, high coverage, and sufficient in scope) outbreak SIAs (oSIAs) could quickly stop transmission, while low quality oSIAs could lead to higher numbers of cases and much wider spread ^16– 19^. Modeling that explored the risks associated with OPV2 cessation demonstrated the importance of preventive increases in population immunity using type 2 containing OPV prior to its globally coordinated cessation ^20^. Modeling also assumed that countries would promptly detect any cVDPV2 outbreaks identified after OPV2 cessation and respond to them as emergencies to aggressively shut them down ^21^. Additional modeling also demonstrated the expected increasing vulnerability of countries to the reintroduction of transmission following importations of type 2 live polioviruses after OPV2 cessation as a function of time ^22, 23^. This work implied the need to fully end all transmission of type 2 live polioviruses within 4 to 5 years after OPV2 cessation, and assumed that in the event of failure, the GPEI would ask manufacturers to restart the production of OPV2 and coordinate the restart of OPV2 use in RI, presumably as trivalent OPV (tOPV) ^6, 18, 24– 27^. In the absence of a well-defined criterion or set of criteria and a process for judging OPV2 cessation failure, or a formal process designed to identify a contingency strategy to restart OPV use in RI, pre-OPV2 cessation modeling used a case-based cumulative incidence trigger and estimated a 6% chance of needing to restart OPV2 in RI, even if the GPEI and countries implemented all of the optimal risk management strategies identified by the model ^18^. Recognizing the potential challenges of the polio endgame, additional pre-OPV2 cessation modeling provided health economic support for investments in the development of new poliovirus vaccine strains, including novel OPV (nOPV), as a contingency plan ^28^. 

As the situation has evolved since 2016, modeling characterized the dynamic risks and logistics of OPV2 restart in RI, and we continued to assume a fixed cumulative case threshold after OPV2 cessation as a trigger for an OPV2 restart ^6, 18, 24– 27^. Modeling that explored the risks associated with OPV cessation demonstrated that increasing population immunity using OPV preventive, planned SIAs prior to OPV2 cessation prevented cVDPV2s in most countries ^29^. However, modeling also highlighted that the observed oSIA performance after OPV2 cessation in many areas was worse ^27, 29^ than pre-OPV2 cessation modeling recommended ^21^. Epidemiological studies similarly indicated continuing cVDPV2 transmission in countries that failed to meet post-cessation oSIA standard operating procedures and implementation milestones related to timeliness and coverage targets ^30^. Following the observed experiences with cVDPV2 transmission in 2019 ^30^, subsequent modeling that incorporated the actual experience of OPV2 cessation risk management efforts found a much higher likelihood of needing to restart OPV2 ^24^. In late 2018, around the time of the 700 ^th^ cumulative reported cVDPV2 case since OPV2 cessation in early 2016, the GPEI signaled to manufacturers the need to restart the production of bulk mOPV2 for use in outbreak response campaigns ^31^. In 2019, the GPEI released its 2019–2023 strategic plan ^32^ that included among other strategies to address VDPV emergence challenges. The primary strategy focused on the accelerated development of type 2 nOPV (nOPV2), which promised more genetic stability and lower risk of seeding new cVDPVs than Sabin mOPV2 for use in outbreak responses ^33^. 

In May 2020, the GPEI released an addendum to the 2019–2023 strategic plan related to cVDPV2 management ^34^ that anticipated widespread use of nOPV2 following its anticipated emergency use listing (EUL). Notably, the 2020 addendum assumed that widespread use of nOPV2 and ideal performance would lead to future reporting that “Novel OPV2 completely replaces Sabin OPV2” and “cVDPV2 outbreaks stopped; new emergence ceased” in 2021 [ 34, page 5]. The addendum also noted the contingency that “if nOPV2 was delayed or in short supply,” then “continue Sabin OPV2 use for outbreak response” or “if needed, restart of OPV2 in preventative campaigns” or in RI would occur [ 34, page 5].

Modeling of the 2019–2023 GPEI strategic plan prior to the COVID-19 pandemic found the polio endgame off-track with respect to ending the transmission of type 1 WPV (WPV1) and cVDPV2s ^24, 35, 36^ by the target dates specified in the plan ^32^. Following the development and early use of nOPV2, a modeling study published in November 2020 suggested that using nOPV2 only to respond to outbreaks would not stop global cVDPV2 transmission ^37^, and anticipated that delays associated with nOPV2 availability would further complicate outbreak response vaccine choices. Subsequent modeling considered the disruptions in polio immunization activities and surveillance caused by the COVID-19 pandemic ^38^ and demonstrated the health consequences of delaying oSIAs ^39^.

In 2021, the GPEI released a strategic plan for 2022–2026, with additional consideration of the role of nOPV2 ^40^. This updated strategic plan anticipates stopping WPV and cVDPV2 transmission by end of 2023 and performing globally-coordinated cessation of bivalent OPV (bOPV, containing types 1 and 3) by 2027 following global certification of all WPV eradication and cVDPV elimination ^40^. Recent modeling of the polio endgame continues to highlight the consequences of weak or suboptimal oSIA performance and reported essentially no chance of successfully stopping all cVDPV2 transmission by 2026 following the current polio endgame trajectory ^41^. However, this recent modeling did not include OPV2 restart in RI since the 2022–2026 GPEI plan does not consider this possibility in any detail ^40^. As the GPEI partners consider the options for the polio endgame strategy going forward, and countries consider their vaccine options for responding to cVDPV2 outbreaks, we explore the complex choices and some logistics associated with restarting the use of OPV2 in RI as well as future OPV use.

## Methods

Anticipating the need for structured discussion of options related to OPV restart as part of the process to develop readiness criteria for potential bOPV cessation, we searched PubMed® for publications related to various combinations of the concepts OPV/OPV2 restart and OPV/OPV2 cessation. We identified a total of 89 publications, a manual review of which found that the only publications that reported prospective modeling of OPV restart into RI as a concept or discussed specific criteria, quantitative triggers, or logistics for OPV restart included the last author of the present manuscript (KMT). We note that the OPV cessation literature includes additional modeling related to the instability of polio eradication after OPV cessation (e.g, 42– 44), which imply the potential need to restart OPV use due to instability in polio eradication after OPV cessation, but do not go further. We reviewed and expanded on the published OPV restart literature to provide a structured discussion of potential options considering risks and triggers. We then developed updated decision trees of polio immunization options ^2– 6^ to provide a visual summary.

## Identification of options considering risks and triggers

The variable nature of transmissibility and neurovirulence of the different poliovirus types (i.e., 1, 2, and 3) and strains (i.e., WPVs, Sabin OPVs, nOPVs, and OPV-related viruses, including VDPVs) influence their behavior and risks ^7, 8, 45^. In addition, the different timing of the cessation of indigenous WPV transmission for each type influences the potential (and actual) timing of phased OPV cessation. Type 3 OPV (OPV3) cessation could theoretically occur any time given the last WPV3 cases reported in 2012 and certification of the eradication of its indigenous transmission in 2019 ^46^. The potential timing of type 1 OPV (OPV1) cessation remains uncertain given ongoing transmission of indigenous WPV1 to date and unknown timing of global certification of its eradication ^47^.

Successful OPV cessation depends on achieving high population immunity to transmission prior to coordinated cessation of the OPV type ^20^. Low population immunity to transmission at the time of withdrawal of type-specific OPV increases cVDPVs risks, which vary by type ^29, 45^. Now 7 years after OPV2 cessation, outbreak response efforts still have not stopped all type 2 live poliovirus transmission in all areas, mucosal population immunity continues to decline in areas with no recent OPV2 oSIAs, and the probability of needing to restart production and broader use of OPV2 to interrupt outbreaks continues to increase ^13, 24, 37^. As we learned from the current type 2 situation and prospective modeling of bOPV cessation, the potential likelihood of restarting OPV1 and/or OPV3 after their coordinated cessation will depend on the management of population immunity to transmission – including in hard-to-reach communities – in the run up to bOPV cessation ^48– 51^. Specifically, the absence of type 3 WPVs since the end of 2012, ongoing cases of VAPP caused by OPV3, the occurrence of some type 3 cVDPV cases, and concerns about OPV production capacity may at some point motivate earlier OPV3 cessation than OPV1 cessation (i.e., phased cessation of the last 2 types) ^50, 51^ despite the absence of this possibility in the current GPEI strategic plan ^40^. Implementation of this option would require the development of sufficient supplies of mOPV1 to replace the bOPV currently used in RI.

After coordinated cessation of one or both of the other OPV types, a decision to restart OPV would not come lightly. The actual decision to restart OPV could require a World Health Assembly resolution and policy documents that recommend OPV restart with statements that the reestablished transmission of a given type of poliovirus cannot be managed or stopped without the reintroduction of type-specific OPV into RI. This could require determining specific criteria and/or a trigger for deciding whether, when, where, and/or how to restart OPV. Early pre-OPV2 cessation modeling of the polio endgame used a threshold of 50,000 total paralytic polio cases of all three types as a criterion for restarting tOPV, while assuming optimal risk management (i.e., minimal emergence risks and very high program performance) by countries and the GPEI, and used a straightforward approach to model OPV restart (i.e., resumption of tOPV use and abandoning OPV cessation as a global strategy to return to control upon reaching the threshold) ^18^. The study also explored other threshold values in sensitivity analyses (i.e., 1,000, 5,000, 10,000, and 15,000 total paralytic polio cases of all three types) ^18^. Post-OPV2 cessation modeling of the polio endgame used a type-specific threshold of 5,000 total paralytic polio cases for restarting OPV for each type, while assuming more realistic risk management and restart strategies that maintained the assumption of continuation of OPV cessation as a global strategy ^24, 35^. Given the current conditions of over 2,900 reported cVDPV2 cases since OPV2 cessation (as of March 7, 2023) ^52^ and more than 1.25 million OPV2 doses released in response to cVDPV2 outbreaks (i.e., over 831 million mOPV2 and tOPV doses and over 450 million nOPV2 doses), current performance of polio eradication efforts, and the development of nOPV and combination IPV products in the polio endgame set of vaccine options, we recognized the need to revisit and expand model assumptions for OPV restart.

Choosing appropriate criteria and/or a trigger for deciding whether to restart OPV in RI poses several challenges. Possible trigger options could include: (i) serotype-specific thresholds of cumulative paralytic polio cases since type-specific OPV cessation (i.e., “case thresholds”), (ii) number of countries/regions affected by transmission or the subset of those with re-established poliovirus transmission (i.e., lasting 12 or some other specified number of months) since OPV cessation (i.e., “spread threshold”), (iii) number of years with ongoing poliovirus transmission since OPV cessation (i.e., “elimination deadline”), or (iv) number of vaccine doses used for outbreak response or needed for the stockpile (i.e., “dose threshold”). While triggers may offer an operational option in terms of modeling, each of them individually is indiscriminate in terms of the others. For example, “case thresholds” ignore the transmission duration and geographical spread, a “spread threshold” may ignore the magnitude of cases and/or the transmission duration, an “elimination deadline” ignores the magnitude of cases and their geographical spread, and a “dose threshold” may ignore all of these but may factor in cost-trade-offs and/or vaccine production capacity constraints. A combination of criteria might represent the best option to match any actual criteria for OPV restart that may emerge. In addition, although we focus on triggers related to modeling, we recognize the OPV restart trigger could be applied to the number or fraction of countries that meet specific criteria for OPV restart in RI and/or a global or regional declaration of established endemic type 2 poliovirus transmission. Some prior modeling demonstrated correlations between some of the different trigger outcomes (e.g., cases and spread of transmission into different areas positively correlate) ^41^. Recent modeling also suggests that transmission may quickly become impractical to control with vaccine production resources available for 2022–2026, if importation of polioviruses occurs into high transmission blocks that represent settings like India or Bangladesh ^53^. However, regardless of the chosen combination, all of these triggers require an explicit choice of their magnitude (i.e., the number of cases, regions/countries effected, years of transmission, and/or doses, or alternative criteria trigger action). 

## Decision trees for immunization programs considering OPV restart

Given the current variety of national polio vaccine schedules that we broadly categorize as IPV-only, sequential (i.e., IPV/OPV), or primarily OPV-using countries that recently added one dose of IPV at the same contact as the third OPV dose (i.e., OPV+IPV) ^35^, we assume that countries and regions would likely make different choices about whether and how to use OPV in their national immunization programs in the event of an OPV restart. A separate study discussed updated polio RI options for the polio endgame that excluded OPV2 restart ^13^, but updated a 2019 discussion ^6^ by adding consideration of schedules that include a minimum of two doses of IPV in RI ^54^. In previous modeling of OPV restart, we did not consider all of the potential vaccine options if nOPV for any or all types becomes available and licensed for use in RI. In Table 1, we list the possible combinations of options of OPV formulations that include 1, 2, or 3 types (i.e., mono-, bi-, or tri-valent OPV formulation) using a notation that identifies the vaccine as OPV followed by the type(s) in the formulation. Table 1 indicates potential Sabin and novel OPV options for each formulation. For any of the OPV formulations that include more than one poliovirus type, co-administration of the doses is represented using a “+”, and separate administrations (when co-administration is not possible) using a “/”. For nOPV doses and co-administration of nOPV in combination with either Sabin or other nOPV types, studies will need to be performed to support licensure and to characterize the per dose effectiveness for each type. For example, these studies could potentially eliminate specific co-administration options in the near future until other products become licensed. This need for specific studies applies to all OPV using combinations that do not involve previously licensed products (e.g., tOPV). 

**Table 1.** Vaccine options for different restart types.

OPV formulation	Potential vaccine options for each OPV formulation
**OPV1**	mOPV1 nOPV1
**OPV2**	mOPV2 nOPV2
**OPV3**	mOPV3 nOPV3
**OPV12**	bOPV(S1,S2) or mOPV1/+mOPV2 bOPV(S1,N2) or mOPV1/+nOPV2 bOPV(N1,S2) or nOPV1/+mOPV2 bOPV(N1,N2) or nOPV1/+nOPV2
**OPV13**	bOPV (S1,S3) or mOPV1/+mOPV3 bOPV(N1,S3) or nOPV1/+mOPV3 bOPV(S1,N3) or mOPV1/+nOPV3 bOPV(N1,N3) or nOPV1/+nOPV3
**OPV23**	bOPV(S2,S3) or mOPV2/+mOPV3 bOPV(N2,S3) or nOPV2/+mOPV3 bOPV(S2,N3) or mOPV2/+nOPV3 bOPV(N2,N3) or nOPV2/+nOPV3
**OPV123**	tOPV or bOPV/+mOPV2 or mOPV1/+mOPV2/+mOPV3 tOPV(N1,S2,S3) or nOPV1/+bOPV(S2,S3) or nOPV1/+mOPV2/+mOPV3 tOPV(S1,N2,S3) or nOPV2/+bOPV or mOPV1/+nOPV2/+mOPV3 tOPV(S1,S2,N3) or nOPV3/+bOPV(S1,S2) or mOPV1/+mOPV2/+nOPV3 tOPV(S1,N2,N3) or mOPV1/+bOPV(N2,N3) or mOPV1/+nOPV2/+nOPV3 tOPV(N1,S2,N3) or mOPV2/+bOPV(N1,N3) or nOPV1/+mOPV2/+nOPV3 tOPV(N1,N2,S3) or mOPV3/+bOPV(N1,N2) or nOPV1/+nOPV2/+mOPV3 tOPV(N1,N2,N3) or nOPV1/+nOPV2/+nOPV3

Abbreviations: OPV, oral poliovirus vaccine; mOPVk, monovalent type k Sabin OPV, where k=1,2 or 3; nOPVk, monovalent type k novel OPV, where k=1,2 or 3; bOPV, bivalent types 1 and 3 Sabin OPV; tOPV, trivalent types 1, 2 and 3 Sabin OPV; Nk, novel type k, k=1, 2 or 3; Sk, Sabin type k, k=1, 2 or 3; bOPV(x,y), potential future bivalent types x and y OPV, where x,y=Nk,Sk; tOPV(x,y,z), potential future trivalent types x, y and z OPV, where x,y,z=Nk,Sk; /+ replaces “/” which indicates separate doses (when co-administration is not possible) or “+” that implies co-administered doses

**Figure 1.**
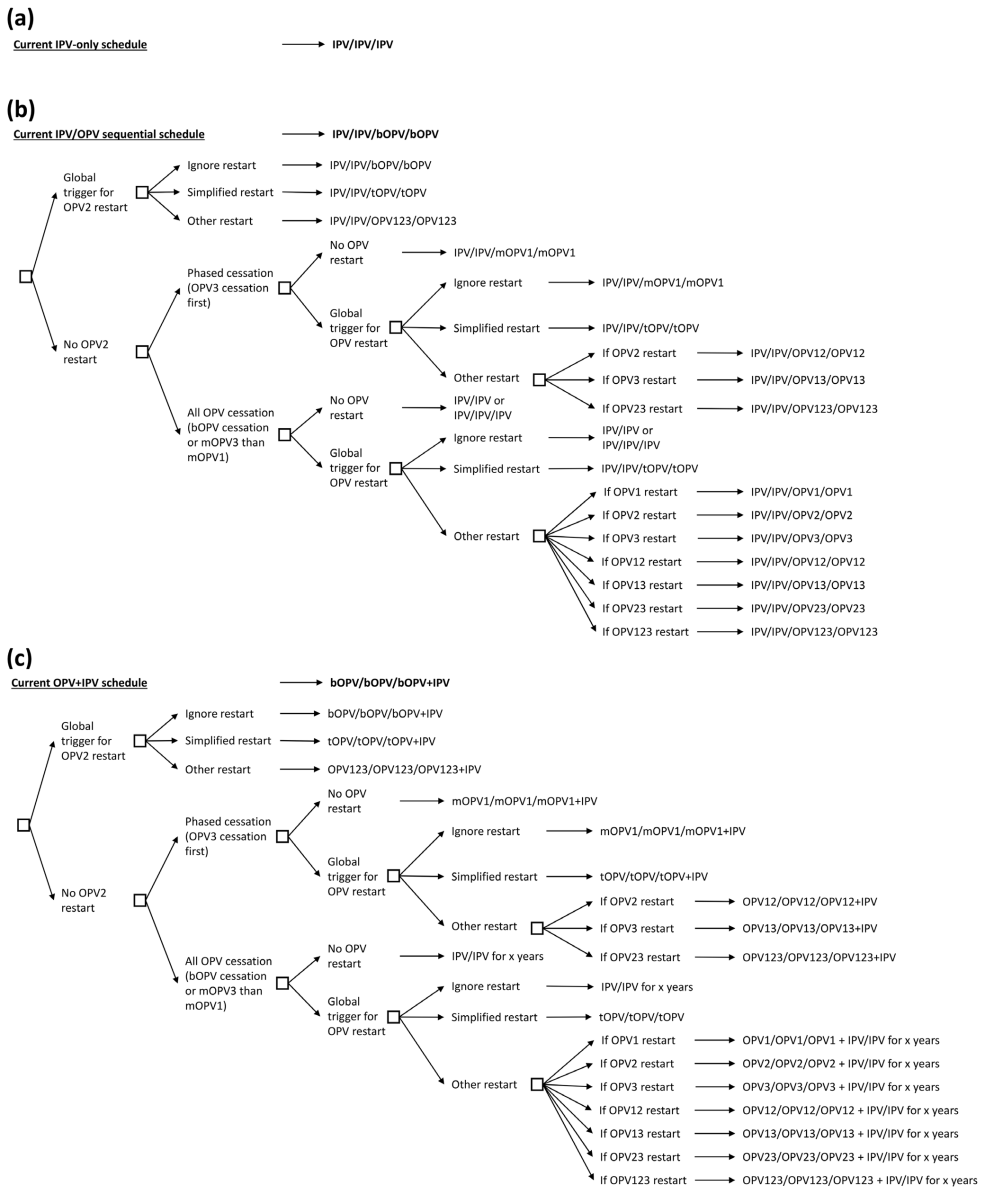
Vaccine schedules for different policies and choices for each current national polio immunization schedule.

Figure 1. shows the vaccine schedules for different policies and choices for each type of current national polio immunization schedule, assuming that those countries currently using only IPV will continue to only use IPV ( Figure 1a). Figure 1a shows a minimum of 3 IPV doses, but actual schedules may include more doses if the country uses IPV as part of a combination vaccine. In the event that an IPV-using country elects to restart the use of OPV, this would shift that country into the IPV/OPV sequential schedule.

For all OPV-using countries, using an IPV/OPV schedule ( Figure 1b) or an OPV+IPV schedule ( Figure 1c), we consider parallel structures using the same simplified categories of broad options that we refer to as “ignore restart,” “simplified restart,” or “other restart.” The latter two OPV options require formulation containing the type(s) already in use in RI combined with the type(s) restarted. The possible options to restart the use of OPV in RI will depend on the type(s) of re-established poliovirus circulation and the stage of OPV cessation process for all three types. OPV cessation itself presents a complicated decision tree on its own ^13^ and the interactions between OPV cessation and restart decisions leads to complex flowcharts ^6^. For the purpose of simplicity, Figure 1 only shows the cessation path that assumes that countries stay on the current national polio vaccine schedules. However, if the national RI schedules changed, we would use the same simplified broad options applied to the new schedules, and we note that individual countries or regions/subregions could decide to shift to a different decision tree. Table 2 shows the differences between the options in Table 1 with respect to the licensure requirements for the potential use of different OPV vaccine formulations in RI as part of OPV restart. We emphasize that licensure would represent a prerequisite for use of any vaccine in RI, and it depends on interest by at least one manufacturer to invest in the required product development and regulatory processes. 

**Table 2.** Factors that may influence the feasibility of different vaccine options.

OPV formulation	Sabin OPV	Novel OPV	Comments
**OPV1**	mOPV1 currently licensed and available	nOPV1 development under way and likely available by the time of potential restart	
**OPV2**	mOPV2 currently licensed and available	nOPV2 currently available under EUL and likely licensed by the time of potential restart	No use of OPV in RI is currently permitted, and declaration of tOPV-bOPV switch failure is a prerequisite for changing this prohibition
**OPV3**	mOPV3 currently licensed and available	nOPV3 development under way and likely available by the time of potential restart	
**OPV12**	Sequential or coadministration of mOPV1 and mOPV2 possible New bivalent formulation of Sabin OPV12 would require licensure	Sequential or coadministration of nOPV1 and nOPV2 would require licensure bnOPV12 would require licensure	Sequential administration of mOPV1 and nOPV2 possible Sequential or coadministration of nOPV1 and mOPV2 would require licensure Mixed Sabin and novel bivalent formulation of OPV12 would require licensure
**OPV13**	bOPV currently licensed and available	Sequential or coadministration of nOPV1 and nOPV3 would require licensure bnOPV13 would require licensure	Sequential or coadministration of mOPV1 and nOPV3 or nOPV1 and mOPV3 would require licensure Mixed Sabin and novel bivalent formulation of OPV13 would require licensure
**OPV23**	Sequential or coadministration of mOPV2 and mOPV3 possible New bivalent formulation of Sabin OPV23 would require licensure	Sequential or coadministration of nOPV2 and nOPV3 would require licensure bnOPV23 would require licensure	Sequential administration of mOPV3 and nOPV2 possible Sequential or coadministration of nOPV3 and mOPV2 would require licensure Mixed Sabin and novel bivalent formulation of OPV23 would require licensure
**OPV123**	tOPV currently licensed and available	Sequential or coadministration of nOPV1 and nOPV2 and nOPV3 would require licensure tnOPV would require licensure	Sequential administration of bOPV and nOPV2 possible Any other mixed Sabin and novel OPV formulation would require licensure

Abbreviations: EUL, emergency use listing; OPV, oral poliovirus vaccine; mOPVk, monovalent type k Sabin OPV, where k=1,2 or 3; nOPVk, monovalent type k novel OPV, where k=1,2 or 3; bOPV, bivalent types 1 and 3 Sabin OPV; tOPV, trivalent types 1, 2 and 3 Sabin OPV; bnOPVx,y, potential future bivalent novel OPV, where x,y={12,23,13; tnOPV, potential future trivalent novel OPV

We first consider the possible options in the context of a decision to restart the use of OPV2 in RI while recognizing that countries may make different choices about whether and how to use OPV in the event of OPV2 restart. If OPV2 restart occurs, individual countries could choose to ignore the OPV2 restart and continue with their current schedule. The “ignore restart” option would also apply to some countries outside of the scope of a limited OPV restart in RI (e.g., regional or subregional restart). Alternatively, in the event of any option to restart OPV in RI, countries could simply replace current bOPV doses with tOPV, which we refer to as a “simplified restart” option. Prior to OPV2 cessation, using Sabin OPV strains as the base for IPV production (i.e., sIPV) ^55^ instead of using more virulent WPV strains, offered the potential to provide a “warm base” for the restart of production of Sabin OPV2 use in RI and re-licensure of tOPV for RI if OPV2 restart became necessary ^19, 26^. If returning to tOPV does not represent a viable option, the “other restart” branch refers to OPV123 formulation options (other than tOPV) listed in Table 1. Different combinations may become appropriate for use and/or available at different points in time, depending on the continued use, cessation, and/or restart of different OPV types. If OPV2 restart does not occur, we assume that individual countries would continue on the path toward cessation of all types of OPV. While at each step of OPV cessation process, any stopped OPV type(s) may require restart of use in RI, and we consider the same categories of the three restart options, with the last one (“other restart”) expanding as the cessation progresses to consider the combinatoric possibilities in Table 1. 

## Discussion

The extensive list of possible OPV formulations raises many research and policy questions, starting with the timing of full licensure of nOPV2, which could allow for potential future use in RI in the event of OPV2 restart. The development and deployment of novel OPV vaccines for type 1 and 3, as well as their respective co-administration possibilities with each other and Sabin OPVs, require additional research to support licensure and to characterize the per dose effectiveness or seroconversion for each type alone and in combinations. In addition to nOPV development, an ongoing effort to introduce hexavalent DTP-Hib-HepB-IPV may add more variations of OPV restart RI schedules and raise the cost of polio vaccination in RI if the number of IPV doses with combination IPV products increases at the same time as the number of OPV doses increases. All efforts to develop new and potential combination vaccines will require substantial investments, and the final products will require attractive pricing for use in RI, particularly for use in low- and lower middle-income countries. Returning to inexpensive Sabin-strain tOPV could pose a preferred option from a health economic perspective ^56^. However, the human cost of annual VAPP and VDPV paralytic cases that motivated OPV cessation may make exercising this option highly unlikely.

Once the safety and effectiveness of nOPV2 is demonstrated in clinical trials and field use, and it might then potentially be used in RI in the event of OPV2 restart, national immunization program leaders may prefer to wait for the availability of nOPV1 and/or nOPV3 prior to bOPV cessation. Such information may be available by the time of potential bOPV cessation, which is planned for 2027 ^40^, or one year after certification of eradication of indigenous WPV1. In addition, because of the substantial operational challenges for program managers associated with stockpile management and distribution of multiple monovalent vaccines, licensing a trivalent nOPV or bivalent nOPV to deliver in combination schedules may be preferred over licensing several monovalent nOPVs, even if only one serotype restart is needed. Therefore, some options in Table 1 are less likely to be implemented than others. In addition to the RI restart options presented, some possibility of using additional innovative polio vaccines always exists, including the potential use of non-replicating seed strains from virus-like particles that are enhanced to provide some higher level of intestinal mucosal immunity after injection ^57– 60^.

Similar to the challenging logistics posed by OPV cessation, the restart of OPV could come with risks that require management. The cessation of OPV2 use in RI in 2016 resulted in at least 7 birth cohorts with no (or limited) exposure to live type 2 polioviruses in countries without OPV2 outbreak responses. In contrast, since 2016, over 35 countries reported cVDPV2 transmission outbreaks and performed OPV2 oSIAs that increased population immunity to transmission for type 2, particularly in large areas of Africa. Arguably, the geographies with sustained, re-established endemic transmission of cVDPV2s despite oSIAs (e.g., Nigeria, Somalia) would represent the logical priorities for the reintroduction of OPV2 in RI, since this may effectively increase population immunity for children served by RI who are close to areas covered by prior oSIAs more rapidly. Any gaps in RI coverage will allow for reservoirs of under-immunized populations to sustain transmission. Since changes in national RI schedules affect all areas, national immunization programs may want to consider a large catch-up SIA for children in all age groups in areas not targeted by oSIAs. 

Mitigation of restart risks requires effective management of vaccine supplies, for example, with accelerated production and development of a stockpile large enough to support coordinated roll-out. However, demands for vaccine for oSIAs would compete with efforts to build up a stockpile to support coordinated RI roll-out, particularly if detection of a cVDPV2 occurs in a high transmission setting with 7 or more birth cohorts with no OPV2 exposure since 2016 ^53^. If restarting OPV2 in RI is urgently considered for certain countries, the use of the large current supplies of mOPV2 prior to their expiration date offers an option to alleviate the demand for OPV2 generated by use in oSIAs and RI ^41^, provided that countries agree to use mOPV2 for restart, which is unlikely. This strategy could deplete the mOPV2 stockpile (with the full benefit of secondary spread of mOPV2), at which point all OPV2 use could shift to nOPV2 ^41^.

Member States have not been sensitized on the possible need for OPV restart and may be reluctant to consider reintroducing OPV2 in their RI schedules, even with nOPV2. However, if countries experience continued transmission and paralytic cases from type 2 polioviruses at higher levels than before OPV2 cessation, perceptions about the relative risks of using OPV2 only for oSIAs (and not in RI) may change. 

As part of the planning for the expected eventual cessation of type 1 and type 3 OPV, it is important to learn from the experience of the OPV2 switch to avoid similar potential challenges in the future. Given the risks of types 1 and 3 VDPV outbreaks and VAPP cases, decision makers may decide against Sabin-strain bOPV cessation without prior replacement with another vaccine. This might lead to demands for development of a bivalent or trivalent nOPV formulation. However, such a path might require many years to complete the needed preclinical development and clinical trials for safety and effectiveness, and most likely would not substantially increase the chances of success on its own ^61^.

Modeling efforts that seek to support vaccine demand forecasts (e.g., 19, 62) face increasing challenges on several fronts. First, forecasts require assumptions about plans to use vaccines, but if decision makers make different choices or take alternative actions, or more generally, if different decision makers design the stockpile than those who use it, this may lead to insufficient supplies and/or wastage due to over supplies. Modeling that forecasted vaccine needs for the mOPV2 outbreak response stockpile assumed that any detections of cases caused by cVDPV2s after 2016 would represent public health emergencies and would lead to aggressive, prompt, high-quality outbreak response activities to shut down the transmission using mOPV2, but this did not occur universally ^39, 63^. Second, forecasts require good assumptions about the performance of the specific vaccines. With nOPV2 use currently occurring only under EUL, and remaining uncertainty about its efficacy in individuals (and effectiveness in populations due to its likely reduced ability to spread secondarily), the implications of the rapid shift in 2021 from oSIAs using mOPV2 to nOPV2 remain uncertain. Modeling of a nOPV2 stockpile must continue exploring bounding assumptions due to remaining uncertainties, but these bounds result in large ranges of vaccine needs. In addition, time for development and clinical trials that theoretically could be needed to demonstrate non-inferiority of different formulations of some combination vaccines in Table 1 provide a substantial hurdle to co-administration of an nOPV with any other mOPV or nOPV. Third, if the cases of cVDPV2s continue to increase and the viruses potentially spread to increasingly vulnerable populations, prospective modeling becomes much more challenging due to the uncertain extent of transmission. Finally, even in the event that forecasters correctly characterize vaccine needs, if the vaccine manufacturers do not produce sufficient quantities of the vaccines for any reason, then the undersupply of vaccines in one time period can create substantial delays in outbreak response. Delays in outbreak response can increase the size and spread of outbreaks, increase ultimate vaccine demand, and exacerbate supply shortages, as has occurred with both mOPV2 and nOPV2 since the switch.

Among the large number of possibilities, numerous factors will likely narrow the set of practical options. Specifically, the timing of consideration of OPV restart relative to the availability of licensed monovalent, bivalent and/or trivalent formulations of nOPV products will affect the set of feasible options considered. The proposed nature of the RI schedule will represent important considerations. Although RI schedules with sequential doses (e.g., bOPV dose followed by nOPV2 at a separate contact) could be adopted for any licensed products, co-administration of different OPV products, if feasible at all, would likely require time for countries and/or manufacturers to evaluate the schedules and/or to formulate products for that specific schedule. Re-formulation leads to issues of fulfilling licensure and regulatory requirements, although changes to schedules with existing licensed products could potentially occur based on addenda to licenses. A decision to restart OPV2 in RI in the near future may look different than when licensed nOPVs for types 1 and 3 and/or combination vaccines containing one or more nOPV may exist. 

## Conclusion

While continued circulation of cVDPV2 since the 2016 switch raises serious questions about the ability of the global polio eradication program to stop cVDPV2 circulation with oSIAs alone, currently there is no established process in place for assessing the need to restart OPV in RI after its cessation. Notably, GPEI did not establish a trigger or threshold for OPV restart prior to the switch. The challenging process of GPEI formally selecting triggers for OPV restart could prove intractable and lead to donor abandonment. Communication of the need for OPV restart as critical to the strategy to reach the ultimate goal of eradication would need to effectively persuade donors and decision makers about implementation of the restart strategy, including the role that international and national vaccine advisory committees would likely play. Future recommendations for RI schedules remain difficult to predict given the current development of numerous products and the evolving epidemiology, but discussions would be very different in the event that OPV restart signals a return to a world that no longer seeks to achieve poliomyelitis eradication by ending all use of OPV. The most important factors narrowing the set of options are limitations in time, national commitment, and financial resources.

## Data Availability

The result of our review rely entirely on reference 6 ( https://doi.org/10.1080/14760584.2019.1635463) such that all of the information generated by this manuscript are reported in Figure 1 - there are no other data to report.

## Reporting guidelines

Standards for Reporting Qualitative Research (SRQR) have been followed.
