# Supplementary material for: Complexity of options related to restarting oral poliovirus vaccine (OPV) in national immunization programs after OPV cessation
Source: Gates Open Res. 2023 Apr 17;7:55. [Version 1] doi: 10.12688/gatesopenres.14511.1 (PMC10403636; doi:10.12688/gatesopenres.14511.1)
Supplement: Peer review history

# Peer review: Reviewer response for version 1

**DOI:** 10.21956/gatesopenres.15825.r34152

**Published:** 2023-08-04

**Authors:** Matt A Price

**Affiliations:** 1 International Aids Vaccine Initiative, New York, New York, USA

The methods would benefit from more details. What key words did you use in your PubMed search? 

 More clarity on what you mean by "risks and triggers" would be welcome. Trigger is presumably a threshold number of new cases or some other event that would inform restarting a polio vaccine campaign that had been previously discontinued, yes? I think I might make that clear somewhere (perhaps I missed it) What's a risk, then?

 In your abstract, you mention documenting the "actual experience" of OPV cessation, but there is no mention of this in your manuscript's methods (did you interview anyone? Did you review reports or discussions of programs as they closed?). What is the ultimate goal of your manuscript? Updating decision trees of when to restart immunizations? 

 "Identification of options considering risks and triggers"  Is this the start of your "results"? This header doesn't seem to mean anything, or at least it was confusing to me. What is this section of your paper attempting to convey to readers? Consider more descriptive header.

 I recommend using fewer acronyms if possible. The paper was a challenging read, and the acronyms did not help.

Is the work clearly and accurately presented and does it cite the current literature?

Yes

If applicable, is the statistical analysis and its interpretation appropriate?

Not applicable

Are all the source data underlying the results available to ensure full reproducibility?

No source data required

Is the study design appropriate and is the work technically sound?

Partly

Are the conclusions drawn adequately supported by the results?

Partly

Are sufficient details of methods and analysis provided to allow replication by others?

No

Reviewer Expertise:

Infectious disease epidemiology and vaccine development

I confirm that I have read this submission and believe that I have an appropriate level of expertise to confirm that it is of an acceptable scientific standard, however I have significant reservations, as outlined above.

# Peer review: Reviewer response for version 1

**DOI:** 10.21956/gatesopenres.15825.r33387

**Published:** 2023-08-04

**Authors:** Ellyn Ogden

**Affiliations:** 1 United States Agency for International Development, Washington, USA

It seems to me the key decisions are related to:   

- To continue with eradication? 
- stay the course with current vaccines but improve SIAs/RI?  
- shift to different
- combos sooner?
- stop efforts to eradicate?  

All of these need triggers.

 Some questions that came to my mind as I was reading your well thought out paper:

1. At what point is the quality of SIAs/RI unable to be of high enough quality to stop transmission? Will a change in vaccine be sufficient to stop transmission and achieve eradication – vaccines don’t deliver themselves – so is quality and coverage the key barrier to eradication not the vaccine formulation? 
2. What criteria would guide the decision to move from an eradication program to a control program?
3. Communicating these options to the governments or communities is daunting – program failures (disguised as vaccine failures) erode trust in vaccination. Explaining cVDPVs is hard enough now, I’m not sure how many reformulations would have to be tested and introduced to get the right combination. This would cause a lot of disruption to health and immunization systems too – country acceptance of ever-changing polio vaccines/EULs/repeated SIAs etc is wavering. With so many options, how many chances does GPEI have to get this right?
4. Manufacturers need 9 - 12 months of advance notice and secured funding just for mOPV1 and, it is unclear how much could be produced and if it would cover large countries..... would/could supply issues drive the decision? If we assume quality of RI and SIAs will remain sub-optimal selecting the right vaccine is important. How much should supply triggers factor into the decisions?
5. Communication with Congress and Parliaments will be tricky - they get nervous when big and expensive mistakes have been made in the past and now, they are being asked to double down on the program.... (Donor agencies with technical staff are less difficult to convince, but still difficult - there are many pressing priorities that can use funds. note: Funds come from appropriators not, generally, donor agencies.) Global messaging has repeatedly said we are on the verge of eradication, nOPV2 is the key to success, polio transition is feasible…. Another decade of experimenting with vaccine formulations without addressing the quality/coverage issues will make it difficult to convince appropriators to stay the course. Why and for how long should donors continue to support this program? Is eradication still feasible?

Questions:

1. I heard that “a recent study led by CDC in Bangladesh (pending publication) focusing on mOPV immunogenicity suggests a higher proportion of type 1 immune response after 1 dose of mOPV1 than bOPV for type 1 at 6 weeks of age. Given this, the PRAG has prepared an analysis on technical aspects of mOPV1 and requested an assessment of the supply implications in case a general switch is made to use mOPV1 for outbreak response. One of the goals of the study is to assess if, in terms of licensed vaccines, bOPV is still the best choice for type 1 outbreak response or if mOPV1 should be considered instead of bOPV for responding to any type 1 outbreak (WPV1 or cVDPV1) on both Immunological and Ethical grounds.”
2. Further, I heard that the Afghanistan and Pakistan MOH/Polio teams want to conduct a seroprevelance assessment to see what the type specificimmunity is before making any major decisions about vaccine changes, but that this was not agreed by the TAG  Given your paper and the lessons learned and implications of vaccine choices – it seems premature to make a major decision based on the current flawed coverage data and the Bangladesh study.
3. Given the above and the previous rack record of decision-making….is the SAGE the only group that should look at and discuss options (even if a SAGE recommendation is needed to move policies forward?) It seems that there needs to be consultations among a larger group of people and organization before making global decisions such as shifting to mOPV1/other vaccine(s) without a thorough, and independent review. It seems the SAGE failed us pre-switch.  Is there a need for a different / supplemental public consultation? What is the best way to make these complex decisions?

Is the work clearly and accurately presented and does it cite the current literature?

Yes

If applicable, is the statistical analysis and its interpretation appropriate?

Yes

Are all the source data underlying the results available to ensure full reproducibility?

Yes

Is the study design appropriate and is the work technically sound?

Yes

Are the conclusions drawn adequately supported by the results?

Yes

Are sufficient details of methods and analysis provided to allow replication by others?

Yes

Reviewer Expertise:

I have managed USAID's polio eradication efforts globally since 1997 and considered an SME in polio eradication.

I confirm that I have read this submission and believe that I have an appropriate level of expertise to confirm that it is of an acceptable scientific standard, however I have significant reservations, as outlined above.

# Peer review: Reviewer response for version 1

**DOI:** 10.21956/gatesopenres.15825.r34162

**Published:** 2023-07-25

**Authors:** Stanley Plotkin

**Affiliations:** 1 University of Pennsylvania and Children's Hospital of Philadelphia, Pennsylvania, USA

This is an important paper as it documents the difficulties of concluding the eradication of paralytic polio owing to the use of a vaccine that itself may cause polio. Despite the success of eradicating types 2 and 3 wild polio the difficulty of concluding eradication of wild type 1 and coping with the spread of mutant type 2 polio (vaccine-associated paralysis and vaccine-derived polioviruses) has delayed eradication for many years and threatens to prevent complete eradication of polioviruses. The conundrum is that only vaccination will eradicate polio but vaccination itself may cause polio. The idea was that outbreaks occurring after cessation of vaccination would initiate emergency vaccination. However, such reintroduction of poliovirus might itself lead to more polio. The development of further attenuated type 2 is a partial answer to the problem, but also might lead to lesser immunization of vaccinee contacts. Current performance of type 2 vaccines does not give optimism about VDPV elimination. Added to that is the current persistence of wild type 1.

 Aside from the uncertainty regarding type 1, OPV2 in various forms continues to circulate and type 3 VAPP cases continue to occur.  Thus the situation regarding eradication is uncertain and the authors state that “transmission may quickly become impractical to control with vaccine production resources available for 2022-2026.” The authors point out that if current strategy fails to produce eradication there is no plan as to how to restart and where to restart OPV immunization. Failure to achieve eradication with current plans might lead to the abandonment of eradication in favor of continued use of OPV or some combination of IPV and OPV. 

 This paper in effect points out the continued desire to eradicate polio together with inadequate consideration of the mutability of the viruses and the large number of asymptomatic infections, leads to indefinite postponement of  polio eradication. The issues raised in this paper have dogged the field over the last 30 and more years and must be dealt with in order that production of both OPV and IPV be commensurate with achievable goals with regard to polio eradication or at least control of the disease.

Is the work clearly and accurately presented and does it cite the current literature?

Yes

If applicable, is the statistical analysis and its interpretation appropriate?

I cannot comment. A qualified statistician is required.

Are all the source data underlying the results available to ensure full reproducibility?

Yes

Is the study design appropriate and is the work technically sound?

Yes

Are the conclusions drawn adequately supported by the results?

Yes

Are sufficient details of methods and analysis provided to allow replication by others?

Yes

Reviewer Expertise:

Vaccines

I confirm that I have read this submission and believe that I have an appropriate level of expertise to confirm that it is of an acceptable scientific standard.
